# Topical Rapamycin as a Treatment for Fibrofolliculomas in Birt-Hogg-Dubé Syndrome: A Double-Blind Placebo-Controlled Randomized Split-Face Trial

**DOI:** 10.1371/journal.pone.0099071

**Published:** 2014-06-09

**Authors:** Lieke M. C. Gijezen, Marigje Vernooij, Herm Martens, Charlene E. U. Oduber, Charles J. M. Henquet, Theo M. Starink, Martin H. Prins, Fred H. Menko, Patty J. Nelemans, Maurice A. M. van Steensel

**Affiliations:** 1 Department of Dermatology, GROW School for Oncology and Developmental Biology, Maastricht University Medical Centre^+^, Maastricht, The Netherlands; 2 Department of Dermatology, VU University Medical Centre, Amsterdam, The Netherlands; 3 Department of Epidemiology, CAPHRI Research School for Public Health Primary Care, Maastricht University Medical Centre^+^, Maastricht, The Netherlands; 4 Department of Clinical Genetics, VU University Medical Centre, Amsterdam, The Netherlands; 5 Department of Clinical Genetics, GROW School for Oncology and Developmental Biology, Maastricht University Medical Centre^+^, Maastricht, The Netherlands; 6 Institute of Medical Biology, Singapore, Singapore; Federico II University, Naples, Italy

## Abstract

**Background:**

Birt-Hogg-Dubé syndrome (BHD) is a rare autosomal dominant disorder characterised by the occurrence of benign, mostly facial, skin tumours called fibrofolliculomas, multiple lung cysts, spontaneous pneumothorax and an increased renal cancer risk. Current treatments for fibrofolliculomas have high rates of recurrence and carry a risk of complications. It would be desirable to have a treatment that could prevent fibrofolliculomas from growing. Animal models of BHD have previously shown deregulation of mammalian target of rapamycin (mTOR). Topical use of the mTOR inhibitor rapamycin is an effective treatment for the skin tumours (angiofibromas) in tuberous sclerosis complex, which is also characterised by mTOR deregulation. In this study we aimed to determine if topical rapamycin is also an effective treatment for fibrofolliculomas in BHD.

**Methods:**

We performed a double blinded, randomised, facial left-right controlled trial of topical rapamycin 0.1% versus placebo in 19 BHD patients. Trial duration was 6 months. The primary outcome was cosmetic improvement as measured by doctors and patients. Changes in fibrofolliculoma number and size were also measured, as was occurrence of side effects.

**Results:**

No change in cosmetic status of fibrofolliculomas was reported in the majority of cases for the rapamycin treated (79% by doctors, 53% by patients) as well as the placebo treated facial sides (both 74%). No significant differences between rapamycin and placebo treated facial halves were observed (p = 1.000 for doctors opinion, p = 0.344 for patients opinion). No significant difference in fibrofolliculoma number or change in size of the fibrofolliculomas was seen after 6 months. Side effects occurred more often after rapamycin treatment (68% of patients) than after placebo (58% of patients; p = 0.625). A burning sensation, erythema, itching and dryness were most frequently reported.

**Conclusions:**

This study provides no evidence that treatment of fibrofolliculomas with topical rapamycin in BHD results in cosmetic improvement.

**Trial Registration:**

ClinicalTrials.gov +NCT00928798

## Introduction

Birt-Hogg-Dubé syndrome (BHD, MIM #135150) was first described in 1975 by Hornstein and Knickenberg and again in 1977 by Birt, Hogg and Dubé [1], [2]. It is a rare autosomal dominant disorder characterized by the occurrence of benign, mostly facial, hair follicle tumours called fibrofolliculomas (FF)s [2], multiple lung cysts, spontaneous pneumothorax [3], and an increased renal cancer risk [4]–[6]. The FFs can be quite disfiguring and are often the reason why patients come to medical attention. FFs occur in around 80% of patients and usually appear after the age of 20 years as dome-shaped, whitish or skin-coloured papules. Typically presenting around the nose, they can also occur on the ears, neck and trunk [2]. Although they do not grow beyond 3–4 mm in size, their numbers increase with age. Consequently, patients can eventually have hundreds of papules that may cause emotional distress and have a strong impact on their quality of life.

Current treatment options for FFs include destructive approaches such as ablative laser, electrocoagulation, excision and other invasive interventions [7]–[10]. These treatments have high recurrence rates (for laser therapy it is known that FFs recur after 2–3 years or even after months [9]) and are not effective in preventing early lesions. Moreover, they have a significant risk of complications such as scarring, inflammation, hypo- and hyperpigmentation. Therefore, there is a medical need for a more targeted therapy that is suitable for chronic use, reduces the number of tumours and/or prevents the growth of new ones. Insights from genetic and cell biological studies have suggested such an approach.

**Figure 1:**
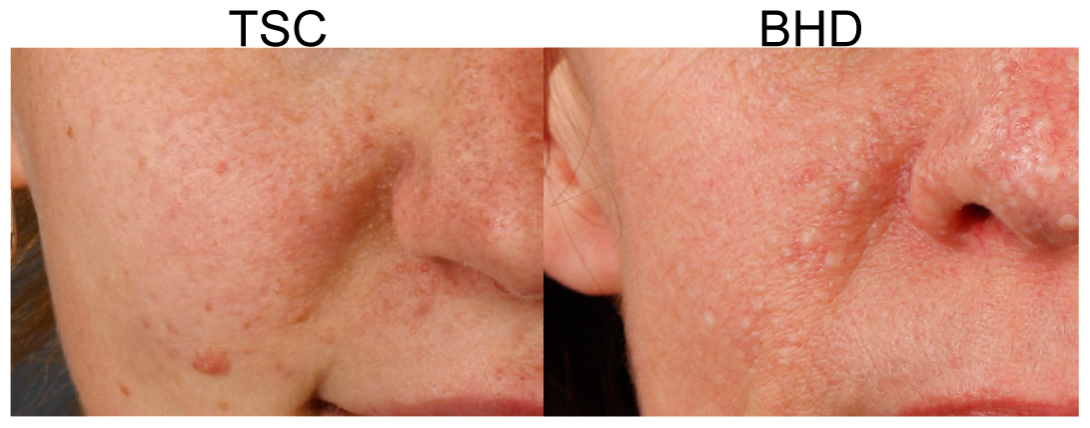
Skin features in TSC and BHD. Photographs taken from a TSC and a BHD patient show angiofibromas and fibrofolliculomas, respectively. Notice the resemblance between the two types of lesion.

BHD syndrome is caused by germline mutations in the *FLCN* gene on chromosome 17p11.2 coding for the protein folliculin (FLCN) [11], [12]. FLCN's functions are mostly unknown, although recent data suggest that it might be involved in cellular energy sensing through the mammalian target of rapamycin (mTOR) signalling pathway [13]. A considerable body of data suggests that in BHD, the mTOR pathway is activated [14]–[18]. Thus, we theorized that BHD syndrome belongs to a larger family of disorders characterized by mTOR deregulation, such as tuberous sclerosis complex (TSC) [14], [19]. In yeast, the homologue of *FLCN* is found to have opposing roles to the genes involved in TSC (*TSC1* and *TSC2*), but all are suggested to regulate common downstream targets [20]. In TSC, as in BHD, patients develop facial hair follicle tumours, called angiofibromas. These tumours strongly resemble FFs and have also been reported in BHD syndrome (Figure 1) [21], [22]. Angiofibromas in TSC were coincidentally found to respond favourably to the mTOR inhibitor rapamycin. They disappeared after only a few months of oral rapamycin, which was administered after renal transplantation [23]. Oral administration of rapamycin has potential side effects that are not acceptable in the context of BHD. However, there are several studies showing that topical application is safe [24]–[26]. Additionally, multiple reports show successful treatment of angiofibromas in TSC with topical rapamycin [24], [26]–[30]. Thus, we hypothesized that rapamycin oral solution might be used for the topical treatment of FFs.

We here show the results of the first double-blind placebo-controlled randomized split-face trial of topical rapamycin for the treatment of FFs in BHD. The objective of this study was to determine whether topical application of rapamycin 1 mg/ml oral solution could lead to overall cosmetic improvement and reduction in size and number of facial FFs in BHD patients. Secondly, we evaluated safety and formula acceptance.

## Materials and Methods

The protocol for this trial and supporting CONSORT checklist are available as supporting information (Protocol S1 & Checklist S1). Before patient recruitment started the study was deposited at ClinicalTrials.gov (NCT00928798; http://www.clinicaltrials.gov/ct2/show/NCT00928798?term=rapamycin+BHD&rank=1).

### Ethics Statement

This study was approved by the Medical Ethical Committees of the Maastricht University Medical Centre^+^ and the VU Medical Centre Amsterdam, Licence number NL28245.068.09 and was conducted in accordance with the Helsinki Declaration of 1975. The individuals shown in this manuscript have given written informed consent (as outlined in PLOS consent form) to publish their pictures.

### Patients

Patient recruitment, inclusion and follow-up occurred at the Department of Dermatology in the Maastricht University Medical Centre^+^ and at the Department of Clinical Genetics in the VU Medical Centre Amsterdam, both reference centres for patients with BHD. Patients with genetically proven BHD, a minimum age of 18 years, at least 10 facial FFs (of which at least one histologically confirmed) and a general good health were eligible for inclusion. Pregnant or lactating women, patients with proven or suspected skin or other malignancy in the past 5 years and patients who planned facial surgery in the treatment period were excluded. Because of the increased risk of renal cell carcinoma in BHD patients, screening by an abdominal ultrasound or MRI was scheduled for patients who had their last screening more than 1 year ago. Patients with concomitant disease requiring systemic immunosuppressive treatment and patients using any facial topical immunosuppressive treatment or facial topical drugs interfering with rapamycin during the trial period or within 30 days before starting therapy were excluded, as were patients with facial skin lesions other than FFs that might worsen under rapamycin, such as active infections. Next to these protocolled criteria, patients were excluded when they could not attend any of the follow-up visits, because this made monitoring of (side) effects and adverse events impossible. A biopsy of a facial FF was taken if this had not been performed earlier. Written informed consent was obtained from all patients eligible for participation.

### Treatment procedures

Computer generated randomisation was performed using random permuted blocks of 2. Randomisation was stratified by participating centres. For each participant, topical treatment with a commercially available rapamycin (sirolimus) oral solution 1 mg/ml was randomized to one facial half and application of a placebo solution to the other facial half. Patients were instructed to topically apply the two formulations twice daily, for 6 months. The rapamycin solution was decanted at 2–8°C in a nitrogen atmosphere and packaged in amber glass bottles to preserve solution quality. The solvent was used as a placebo and was packaged in identical bottles. Bottles were numbered and marked ‘left’ or ‘right’ by the pharmacist, in order to conceal randomisation. Patients and investigator were both blinded for the treatment of each facial side throughout the study.

### Efficacy assessment

Patients were clinically assessed at baseline, 3 months and 6 months by a single investigator (LMCG). Evaluation consisted of careful examination of the face, including assessment of facial FF number and size, as well as the change in general cosmetic FF status compared to baseline. At each visit, facial medical photographs were obtained under standardized conditions concerning: studio lighting, subject positions, predetermined camera settings (distance to subject, resolution, aperture settings, exposure time) and background. For each patient full front, left and right oblique views (45°) and left and right profile views (90°) were obtained. Photographs were evaluated twice by 3 physicians; the investigator and 2 dermatologists. All were blinded for the treatment. They assessed the size of the largest FF in a predefined area. Right and left facial halves were scored separately and 3-month and 6-month photographs were compared to baseline. The change in general cosmetic FF status was evaluated using a 7-point Likert scale (−3 =  strong worsening; −2 =  moderate worsening; −1 =  minimal worsening; 0 =  no change; 1 =  minimal improvement; 2 =  moderate improvement; 3 =  strong improvement) [31], [32]. The FF number was registered in categories, (0; 1–10;11–20;21–30; 31–40; 41–50; >50). Each of the three observers scored the photographs twice for cosmetic status, FF size and FF number. From the six Likert scores or categories obtained per patient the most frequently assigned score was used for analysis. When multiple scores were obtained in equal amounts, the score indicating the least improvement was used. The change in FF number was obtained by comparing the 6-month results to baseline results. Reduction of FF number was defined as a decrease of at least one category. Patients' impressions concerning cosmetic FF status were assessed as well, also using the 7-point Likert scale. At follow-up visits we assessed and registered side effects.

### Statistical analysis

A minimum of 17 patients was needed to detect an increase in preference for rapamycin over placebo by at least 35% (from 50% to 85%), with a power of 90% and a type 1 error (2 sided) of 0.05. A minimum of 35% difference in response was considered to be clinically relevant, as a smaller difference does not justify the routine treatment of BHD patients with rapamycin. The McNemar's chi-square test was used to test for discrepancies in response to rapamycin and placebo treatment concerning cosmetic improvement and reduction in FF number. This test was also used to compare the proportion of side effects occurring between the two facial sides. Differences in FF size were tested for significance using the T-test for paired samples. A p-value smaller than 0.05 was considered to indicate statistical significance in all analyses. The inter-observer variation of scores on cosmetic status, FF size and number was measured using the intraclass correlation coefficient. For data analysis we used SPSS Statistics version 20.0.0.1 (IBM, Armonk, NY, USA) and for calculation of 95% confidence intervals (95%CI) for difference in paired proportions we used R package ExactCIdiff version 2.15.1 (http://cran.r-project.org/web/packages/ExactCIdiff/index.html).

## Results

### Study population

**Figure 2:**
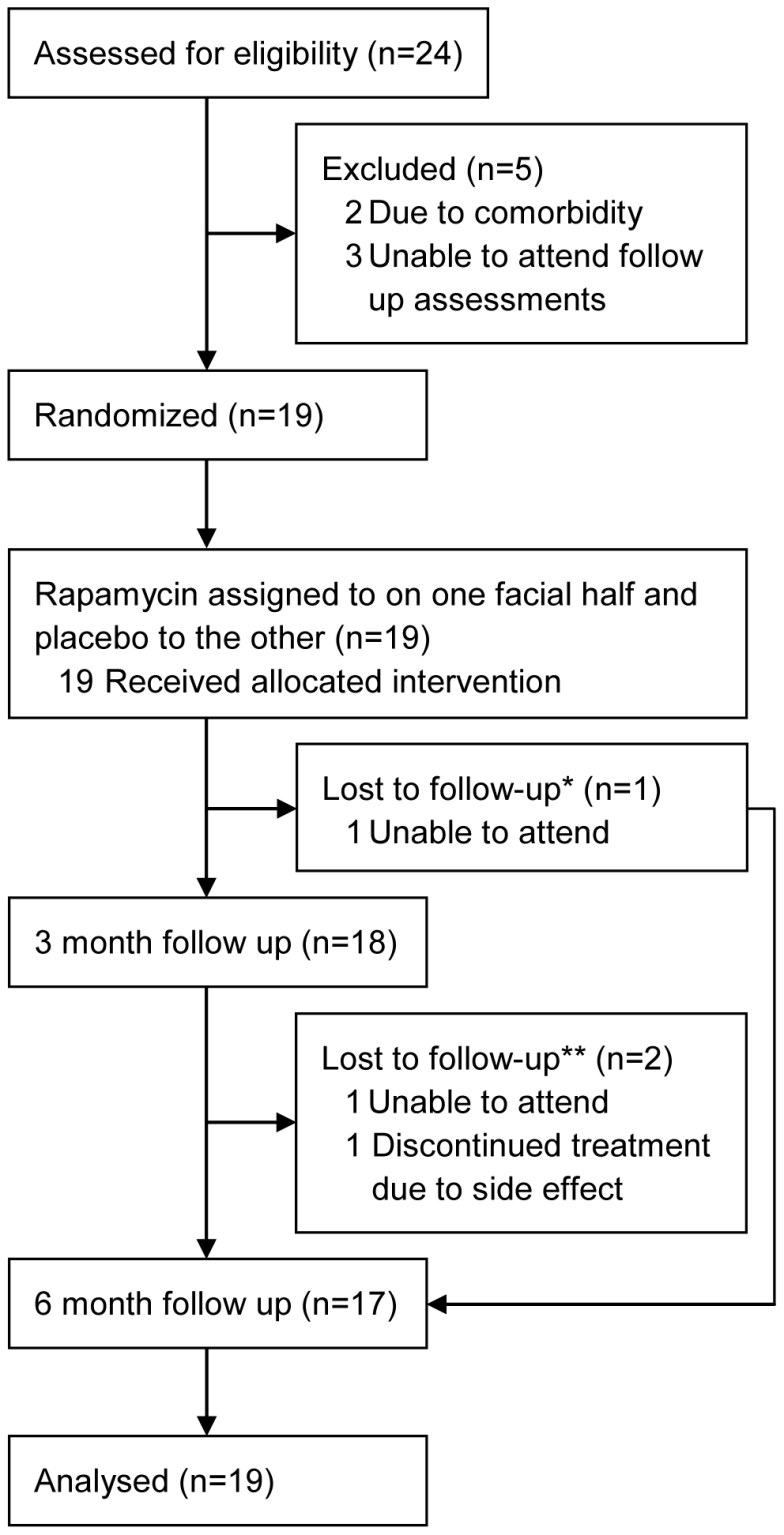
Trial outline. * The patient who was lost to follow-up at 3 months was excluded from the analysis of the 3 month results. ** For the patients who were lost to follow-up at 6 months, the 3-month data were carried forward in the analysis of the 6-month results.

Between January 2010 and August 2010, 24 patients with BHD were recruited and assessed for eligibility to participate (Figure 2). One patient was excluded due to excessive use of alcohol and three because of frequent travelling abroad, which made follow-up impossible. After screening, but before randomisation, one person had to be excluded due to the diagnosis of a malignancy not related to BHD. A total of 19 individuals were randomised. The distribution of baseline characteristics of the study population is shown in table 1. One individual was not able to attend the 3-month follow-up visit due to immobility after back surgery, but could be evaluated at six months. This person was excluded from the data analysis of the 3-month results (n = 18). After two months of treatment, one patient had to discontinue the treatment because of a tearing eye. This patient was seen at the 3-month follow-up visit, but was considered lost to follow-up at six months. A second patient was lost to follow-up at six months due to travelling. For both, the 3-month data were carried forward in the 6-month efficacy analysis (n = 19). An additional analysis of the 6-month results was performed in which all persons that were lost to follow-up at six months were excluded from the analysis (n = 17).

**Table 1:** Baseline characteristics of included patients.

Characteristic		Number of patients (n = 19)
Gender	Male	13
	Female	6
Mean age, years (range)		52 (34–70)
Number of facial fibrofolliculomas	20–50	4
	50–100	4
	>100	11
*FLCN* mutation	c.319_320delGTinsCAC	1
	c.610_611delGCinsTA	9
	c.655dupC	1
	c.1177-2A>G	1
	c.1285dupC	3
	c.1408_1418del	1
	c.871+3_c.871+4delGAinsTCCAGAT	1
	c.880G>T	1
	c.250-?_1740+?del	1
Previous treatment	None	15
	Laser	4
	Surgical	1

### Effects of rapamycin treatment

The effect of rapamycin was assessed by measuring changes in cosmetic status, number and size of the FFs.

**Figure 3:**
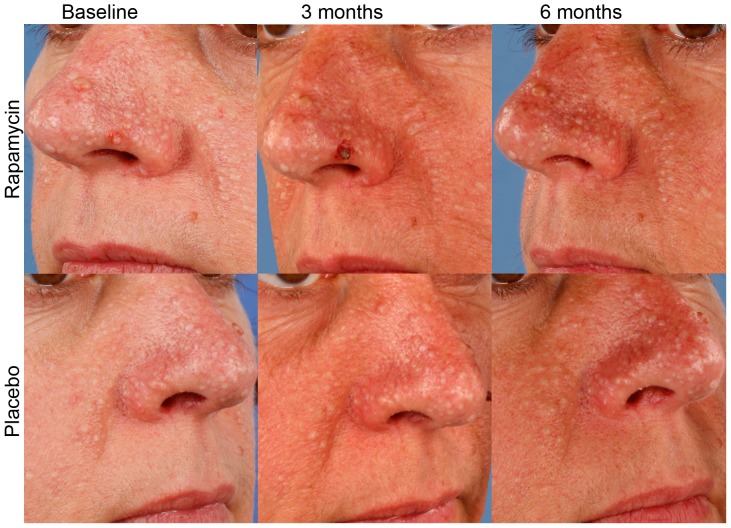
Photographic recording of cosmetic status during rapamycin treatment. Close up of standardised facial photographs taken at baseline, 3 months and 6 months after starting treatment with topical rapamycin or placebo per facial half. Photos of a representative patient are shown, revealing no visible improvement or worsening of the facial fibrofolliculomas.

**Figure 4:**
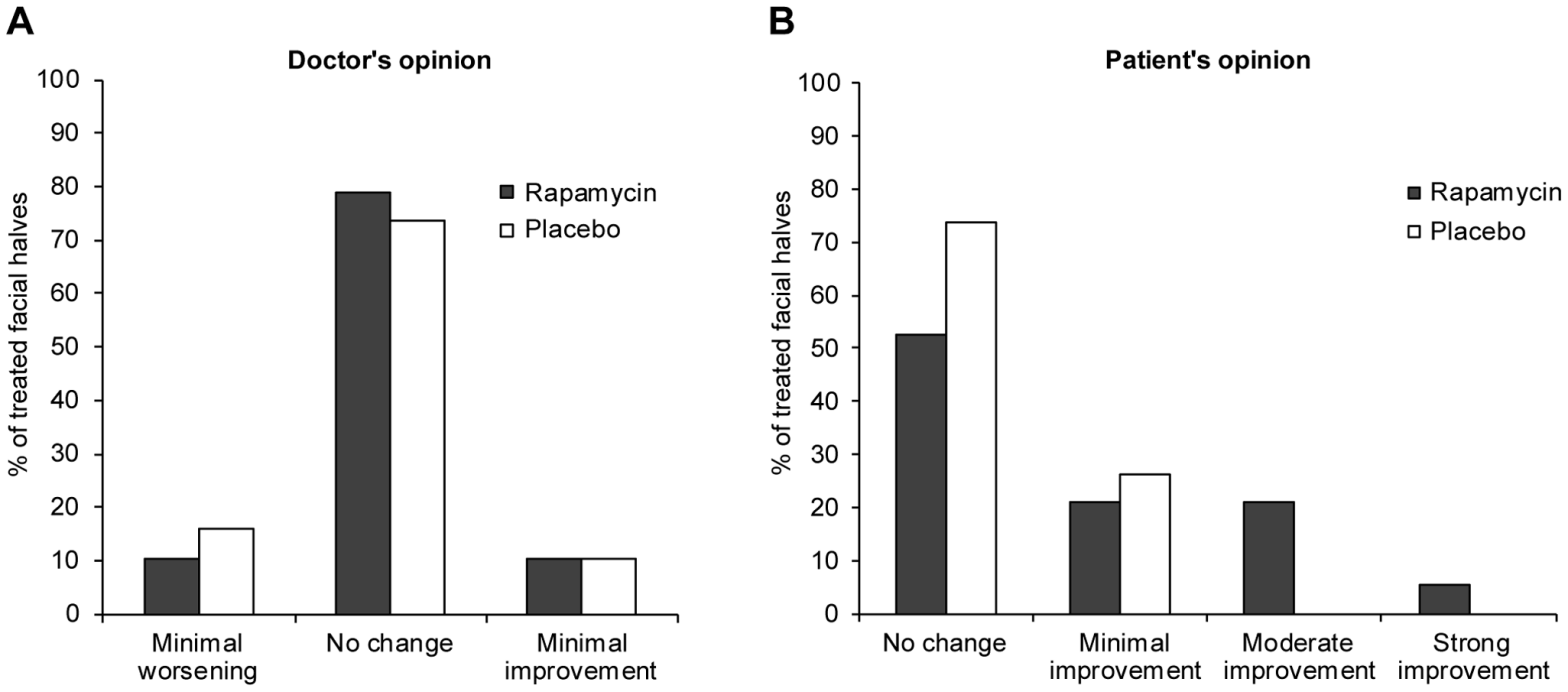
Changes in cosmetic status of fibrofolliculomas after treatment with rapamycin scored by doctors and patients. BHD patients with facial fibrofolliculomas were treated with topical rapamycin and placebo for six months. The degree of improvement compared to baseline was scored on a 7-point Likert scale by both doctors (A) and patients (B). Doctors and patients reported no change in the majority of cases. Patients reported improvement more frequently for the rapamycin compared to the placebo treated facial side.

Cosmetic improvement of the FFs as judged by doctors and patients is presented in figures 3 and 4. There were no significant differences between rapamycin and placebo treated facial sides. According to doctors' opinion, improvement of cosmetic status was seen in 10.5% of rapamycin treated facial halves and in 10.5% of the placebo treated sides. The difference is 0% with a 95%CI of -19% to +19%; p = 1.000 (Appendix S1). Patients reported cosmetic improvement upon rapamycin treatment more often than upon placebo treatment (47.3% versus 26.3%). The difference is 21% with a 95%CI of −14% to +51%; p = 0.344 (Appendix S1).

**Figure 5:**
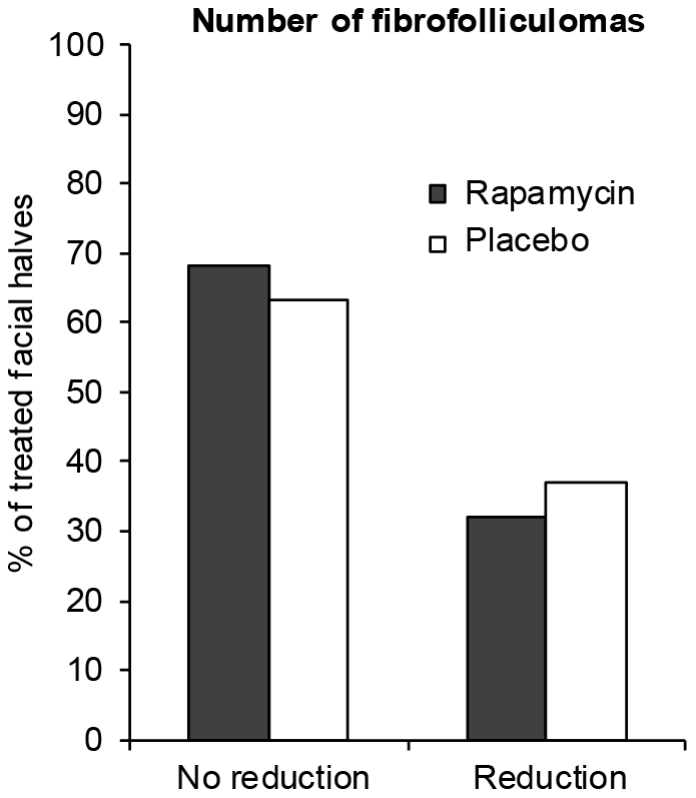
Changes in the number of fibrofolliculomas after treatment with rapamycin. BHD patients with facial fibrofolliculomas were treated with topical rapamycin and placebo for six months. The number of fibrofolliculomas in a predefined area of the face was determined and compared to baseline. The reduction in the number of fibrofolliculomas was stronger upon placebo treatment.

Both doctors and patients reported no changes in the majority of cases for the rapamycin treated (79% and 53% respectively) as well as the placebo treated facial sides (both 74%). The doctors reported minimal worsening in 16% of the placebo treated versus 11% of the rapamycin treated facial halves, whereas minimal improvement was observed in 11% of both rapamycin and placebo treated facial sides (Figure 4). Patients reported improvement more frequently for the rapamycin treated compared to the placebo treated facial side. Nine patients (47%) reported minimal to strong improvement of the rapamycin treated facial half, versus five (26%) reporting improvement of the placebo treated half (Figure 4). Additional analysis of the 17 patients that were seen at the 6-month follow-up showed similar results for the doctors' opinion on cosmetic improvement of FFs after treatment. Reduction in FF number was observed in 32% of rapamycin treated sides versus 37% of placebo treated sides, with a difference of 5% with a 95%CI of −20% to +31%; p = 1.000 (Figure 5 and Appendix S1)

After three months of treatment the mean change in FF size was 0.054 mm on the rapamycin treated sides and 0.027 mm on the placebo treated sides, with a mean difference of 0.027 mm with a 95%CI of −210 to +0.264 mm; p = 0.184 (Table 2).

**Table 2:** Between-treatment comparison of mean change in fibrofolliculoma size compared to baseline (in mm) using the T-test for paired samples.

	Rapamycin	Placebo	Difference	95% CI	p-value
At 3 months	0.054 (SD 0.34)	0.027 (SD 0.27)	0,027	−0,21–+0,26	0.814
At 6 months	0.100 (SD 0.29)	0.096 (SD 0.32)	0.004	−0,16–+0,17	0.961

SD = standard deviation; CI = confidence interval.

The inter-observer variations, expressed as intraclass correlation coefficients (ICC) for cosmetic status, FF number and FF size are shown in Table 3.

**Table 3:** The inter-observer variation of scores used to measure the effect of rapamycin using the intraclass correlation coefficient (ICC).

Outcome measure	Lowest ICC (95%CI)	Highest ICC (95%CI)
Cosmetic status of fibrofolliculomas	0.517 (0.244–0.758)	0.633 (0.390–0.821)
Number of fibrofolliculomas	0.643 (0.324–0.840)	0.878 (0.757–0.947)
Size of fibrofolliculomas	0.374 (0.075–0.660)	0.653 (0.401–0.834)

95%IC = 95% confidence interval.

### Side effects of topical rapamycin

**Figure 6:**
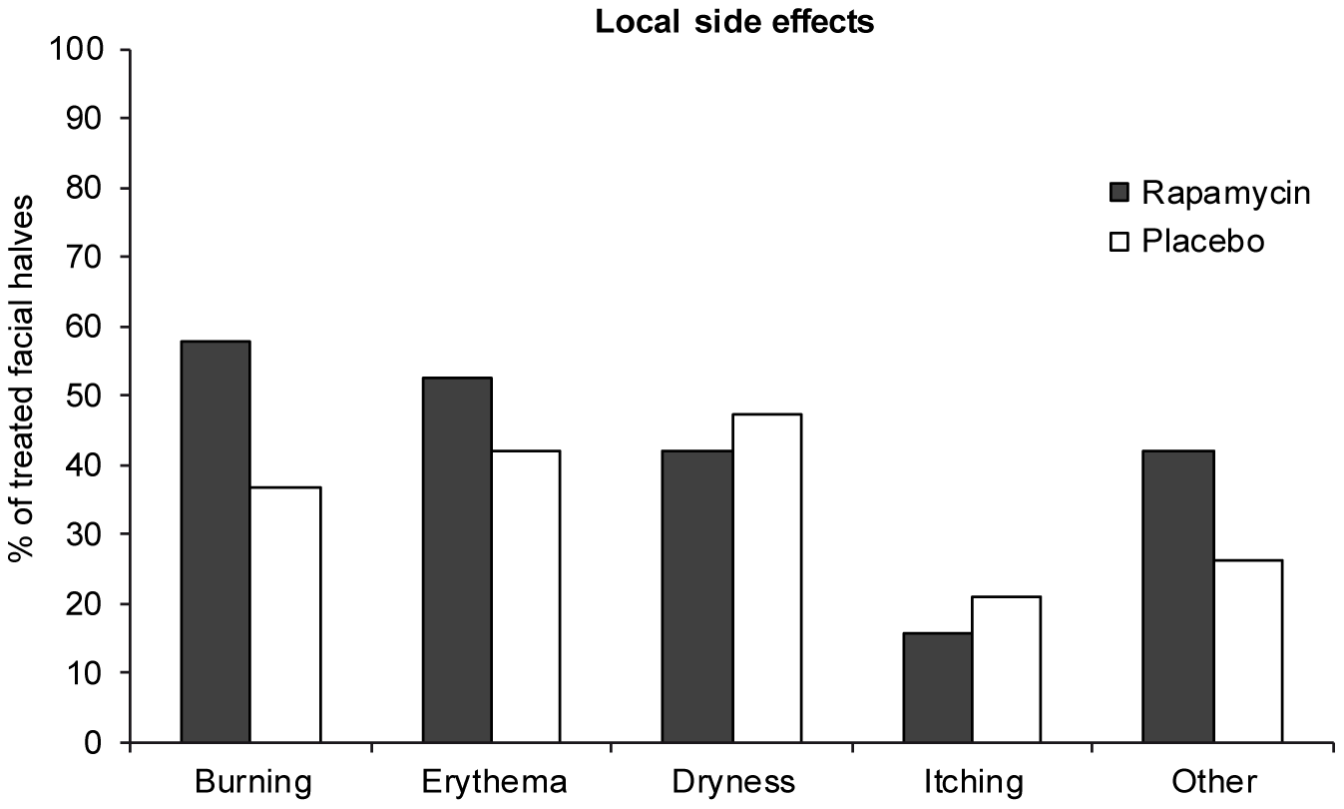
Local side effects of topical rapamycin treatment. Side effects of both topical rapamycin and placebo treatment were registered at each clinical assessment. Burning, erythema, dryness and itching were the most commonly reported local side effects. All side effects other than dryness and itching were reported more frequently for the rapamycin treated facial half.

The majority of patients reported one or more side effects during the six months of treatment for rapamycin (68%) as well as for placebo (58%) (difference 10% with 95%CI −14.3 to 35.0; p = 0.625). A burning sensation, erythema, dryness and itching were most frequently reported (Figure 6). Other side effects were tearing and prickling eyes, scaling, moderate pain and a prickling sensation of the skin. Most side effects occurred in the first three weeks of treatment and resolved over time, with 71% reported at the 3-month follow-up visit and only 42% at the 6-month visit. No serious adverse events have occurred during treatment.

## Discussion

This is the first randomised controlled trial in which the effectiveness of rapamycin on persistence and growth of FFs in patients with BHD was studied. We provide evidence that topical rapamycin does not improve the cosmetic status of FFs in these patients and does not reduce the amount or size of the FFs.

Previous studies in rodent and *Drosophila* models have suggested a role for increased mTOR activity in the pathogenesis of BHD, prompting this study [15]–[17], [33]. However, other mouse models subsequently showed conflicting data concerning the nature of the link between FLCN and mTOR. In contrast to other studies, Hudon et al. and Hartman et al. found decreased mTOR activity in mice [15]–[17], [34], [35]. In human BHD tumours, the role of mTOR had also been questioned by Claessens et al. based on immunohistochemical assessment of BHD patient skin and kidney tumour tissue [36]. More recent *in vitro* studies using *FLCN* knockdown cells have not been able to confirm a direct link between FLCN and mTOR [37] (and unpublished data). The deregulation of mTOR activity found in the tumours of BHD animal models could be due to mTOR's general involvement in cancerous growth rather than a direct effect of FLCN's absence. Of note, most animal models involve knockout of *FLCN*. FFs do not have loss of *FLCN* and we have recently provided evidence that BHD-associated kidney tumours do not have complete loss of *FLCN* either [38]–[40]. Thus, the present state of the art suggests that there is no clear-cut relation between FLCN and mTOR. Our trial data support this assertion and we suggest that mTOR does not play an active role in the maintenance or growth of fibrofolliculomas, in contrast to angiofibromas in TSC.

We have considered alternative explanations for the observed lack of efficacy. Firstly, the concentration of rapamycin in the lotion might have been too low. However, the oral rapamycin solution that we used in this study has been shown to be an effective topical treatment for angiofibromas in TSC, without detectable levels of rapamycin in the serum [26]. A 1∶1 mixture of rapamycin solution 1 mg/ml in an emollient has also shown to be effective in TSC [27]. This indicates that rapamycin in this oral solution penetrates into the skin and that the concentration of rapamycin we used is sufficient to cause a clinical effect. Propylene glycol is part of the solvent of the oral rapamycin and is known to enhance topical drug delivery [41]–[44]. Secondly, patient compliance might have been too low. To measure compliance we planned to weigh the used bottles with rapamycin and placebo solution at every follow-up visit. Because the treated surface varied widely between patients and not all bottles were retrieved, compliance could not be determined in this way. No patient reported to have deviated from the treatment protocol, but we cannot rule out non-compliance. However, as most patients experienced one or more side effects, we are confident that they did indeed adhere to protocol. Thirdly, there was moderate variation between the doctors in scoring the change in cosmetic status and the size of FFs. Small random errors in classification of cosmetic status theoretically may result in obscuring an effect of rapamycin. However, it seems improbable that large, clinically relevant differences would be missed by lack of perfect agreement. Moreover, ICCs up to 0.878 were obtained for observer agreement on FF number. For this outcome measure the results clearly show no advantage of rapamycin treatment compared to placebo treatment.

In this randomised controlled trial both doctors' and patients' opinion on the efficacy of the topical rapamycin treatment of FFs in BHD was measured, giving disparate results. Patients reported improvement in some cases, whereas doctors observed no efficacy of rapamycin treatment. All three doctors were blinded and they evaluated the treatment twice at separate time points. Patients were also blinded at the start of the treatment, but because side effects occurred more often on the rapamycin treated side blinding could have been compromised. Therefore patients might have been biased towards experiencing improvement on the facial half with most side effects. This can explain the difference in doctors' and patients' opinion on improvement of the FFs after treatment.

Most reported side effects were a sign of skin irritation. Although these irritation signs occurred more often on the rapamycin treated side, they were also reported on the placebo treated side of the face. This suggests that the excipient, which contains ethanol, may have been responsible for some of the side effects.

In conclusion, we show that topical rapamycin as used by us is not an effective treatment for FFs in BHD. It is still possible that a higher concentration or oral administration could be effective. However, very recent work provides evidence that reduced expression of FLCN does not necessarily cause increased mTOR activity. Thus, our present findings do not support further study of rapamycin or similar compounds for the treatment of FF in BHD. Development of an effective topical treatment will have to await full elucidation of FLCN's functions in cellular signalling.

## Supporting Information

Appendix S1 **Cross tabulations for cosmetic outcome and fibrofolliculoma number.**
(DOCX)

Checklist S1 **CONSORT checklist.**
(DOC)

Protocol S1 **Trial protocol.**
(DOC)
