# Supplementary material for: Topical Rapamycin as a Treatment for Fibrofolliculomas in Birt-Hogg-Dubé Syndrome: A Double-Blind Placebo-Controlled Randomized Split-Face Trial
Source: PLoS One. 2014 Jun 9;9(6):e99071. doi: 10.1371/journal.pone.0099071 (PMC4049818; doi:10.1371/journal.pone.0099071)
Supplement: Appendix S1 — Cross tabulations for cosmetic outcome and fibrofolliculoma number. (DOCX) [file pone.0099071.s001.docx]

**APPENDIX SI** Cross tabulations for cosmetic outcome and fibrofolliculoma number

The cross tabulations shown here were used to calculate the 95% confidence intervals and p-value for doctors’ and patients’ opinion on cosmetic outcome.

**Doctors’ opinion on changes in cosmetic status**

|  | | Placebo treated facial halves | | |
| --- | --- | --- | --- | --- |
|  | | No improvement | Improvement | Total |
| Rapamycin treated facial halves | No improvement | 17 | 0 | 17 |
|  | Improvement | 0 | 2 | 2 |
|  | Total | 17 | 2 | 19 |

**Patients’ opinion on changes in cosmetic status**

|  | | Placebo treated facial halves | | |
| --- | --- | --- | --- | --- |
|  | | No improvement | Improvement | Total |
| Rapamycin treated facial halves | No improvement | 7 | 3 | 10 |
|  | Improvement | 7 | 2 | 9 |
|  | Total | 14 | 5 | 19 |

**Changes in fibrofolliculoma number**

|  | | Placebo treated facial halves | | |
| --- | --- | --- | --- | --- |
|  | | No reduction | Reduction | Total |
| Rapamycin treated facial halves | No reduction | 10 | 3 | 13 |
|  | Reduction | 2 | 4 | 6 |
|  | Total | 12 | 7 | 19 |
