# Supplementary material for: Topical Rapamycin as a Treatment for Fibrofolliculomas in Birt-Hogg-Dubé Syndrome: A Double-Blind Placebo-Controlled Randomized Split-Face Trial
Source: PLoS One. 2014 Jun 9;9(6):e99071. doi: 10.1371/journal.pone.0099071 (PMC4049818; doi:10.1371/journal.pone.0099071)
Supplement: Protocol S1 — Trial protocol. (DOC) [file pone.0099071.s003.doc]

**Topical rapamycin to treat fibrofolliculomas in Birt-Hogg-Dubé syndrome**

**PROTOCOL**

**04-03-2010**

**TITLE:**  **Topical rapamycin to treat fibrofolliculomas in Birt-Hogg-Dubé syndrome**

| **Protocol ID:** | **BHD_rapa_01** |
| --- | --- |
| **Short title:** | **Topical Rapamycin** |
| **Version:** | **3** |
| **Date:** | **04-11-2009** |
| **Coordinating investigator:** | **Drs. L.M.C. Gijezen**  **Research physician**  **Department of Dermatology Maastricht UMC**  **Email:** [**lieke.gijezen@mumc.nl**](mailto:lieke.gijezen@mumc.nl)  **Tel: 043-3875641** |
| **Principal investigators:** | **Dr. M.A.M. van Steensel**  **Dermatologist**  **Department of Dermatology Maastricht UMC**  **Email:** [**M.van.steensel@mumc.nl**](mailto:M.van.steensel@mumc.nl)  **Tel: 043-3877292**  **Dr. F.H. Menko**  **Clinical Geneticist**  **Department of Clinical Genetics VUMC Amsterdam**  **Email:** [**FH.Menko@vumc.nl**](mailto:FH.Menko@vumc.nl)  **Tel: 020-4440150** |
| **Sponsors:** | **Maastricht University Medical Centre**  **(Maastricht UMC)**  **VU Medical Centre Amsterdam**  **(VUMC Amsterdam)** |
| **Independent physician:** | **Dr. N.W.J. Kelleners-Smeets**  **Dermatologist**  **Department of Dermatology Maastricht UMC**  **Email:** [**N.Kelleners.smeets@mumc.nl**](mailto:N.Kelleners.smeets@mumc.nl)  **Tel: 043-3877295** |
| **Pharmacy:** | **Clinical Pharmacy**  **VUMC Amsterdam** |

**TABLE OF CONTENTS**

1. INTRODUCTION AND RATIONALE 7

2. OBJECTIVES 9

3. STUDY DESIGN 10

4. STUDY POPULATION 12

4.1 Population (base) 12

4.2 Inclusion criteria 12

4.3 Exclusion criteria 12

4.4 Sample size calculation 12

5. TREATMENT OF SUBJECTS 13

5.1 Investigational product/treatment 13

5.2 Use of co-intervention 13

6. INVESTIGATIONAL MEDICINAL PRODUCT 14

6.1 Name and description of investigational medicinal product 14

6.2 Summary of findings from non-clinical studies 14

6.3 Summary of findings from clinical studies 14

6.4 Summary of known and potential risks and benefits 14

6.5 Description and justification of route of administration and dosage 15

6.6 Dosages, dosage modifications and method of administration 15

6.7 Preparation and labelling of Investigational Medicinal Product 15

6.8 Drug accountability 16

7. METHODS 17

7.1 Study parameters/endpoints 17

7.1.1 Main study parameter/endpoint 17

7.1.2 Secondary study parameters/endpoints (if applicable) 18

7.1.3 Other study parameters (if applicable) 18

7.2 Randomisation, blinding and treatment allocation 18

7.3 Study procedures 19

7.4 Withdrawal of individual subjects 20

7.5 Replacement of individual subjects after withdrawal 20

7.6 Follow-up of subjects withdrawn from treatment 20

7.7 Premature termination of the study 20

8. SAFETY REPORTING 21

8.1 Section 10 WMO event 21

8.2 Adverse and serious adverse events 21

8.2.1 Suspected unexpected serious adverse reactions (SUSAR) 22

8.2.2 Annual safety report 22

8.3 Follow-up of adverse events 23

9. STATISTICAL ANALYSIS 24

9.1 Descriptive statistics 24

9.2 Primary analysis 24

9.3 Interim analysis 24

10. ETHICAL CONSIDERATIONS 25

10.1 Regulation statement 25

10.2 Recruitment and consent 25

10.3 Benefits and risks assessment, group relatedness 25

10.4 Compensation for injury 26

10.5 Incentives 26

11. ADMINISTRATIVE ASPECTS AND PUBLICATION 27

11.1 Handling and storage of data and documents 27

11.2 Amendments 27

11.3 Annual progress report 27

11.4 End of study report 28

11.5 Public disclosure and publication policy 28

12. REFERENCES 29

13. ANNEXES……………………………………………………………………………………31

**SUMMARY**

**Rationale:** Birt-Hogg-Dubé syndrome (BHD) is a rare autosomal dominant disorder characterized by the occurrence of benign, mostly facial, hair follicle tumours called fibrofolliculoma, multiple lung cysts and (probably as a result) spontaneous pneumothorax and kidney cancer. The fibrofolliculomas can be quite disfiguring and are usually the reason that patients come to medical attention**.**

A topical treatment for chronic use that reduces the number of tumours and/or prevents the growth of new ones would be preferable but is not yet available.

Research on mouse models and our own human data suggest that in BHD syndrome there is deregulation of mTOR signalling. mTOR, mammalian target of rapamycin, is a multiprotein-complex that is a central player in cellular growth regulation and energy sensing. We think that in BHD the mTOR protein is upregulated or overactivated which causes tumour growth, including fibrofolliculomas. This would mean that mTOR inactivation could prevent growth of fibrofolliculomas. New insights from genetic and cell biological studies now suggest a potential therapy; the mTOR inhibitor rapamycin. Rapamycin oral solution has been used topically in previous studies and was both safe and effective. Thus, we have theorized that rapamycin oral solution might be used safely topically for the treatment of BHD-associated fibrofolliculomas.

**Objectives**: Primary objective: To determine whether topical application of rapamycin can lead to reduction in size and/or number of fibrofolliculomas in BHD patients and may prevent the growth of new ones. Secondary Objectives: Safety, formula acceptance and patient satisfaction.

**Study design:** Double-blind placebo-controlled randomized intervention study.

**Study population:** Otherwise healthy patients ≥ 18 years of age with genetically proven Birt-Hogg-Dubé syndrome and facial fibrofolliculomas.

**Intervention**: Application of rapamycin 1 mg/ml oral solution to one predefined skin area on one facial half, twice daily. The other facial half will be similarly treated with a placebo.

**Main study endpoints:** The main study parameter is significant regression (reduction in facial fibrofolliculoma size and count) in the treated area due to topical rapamycin. Secondary endpoints are the absence of any effect, tolerability, adverse events, formula acceptance and patient satisfaction.

**Nature and extent of the burden and risks associated with participation, benefit and group relatedness:** Participation requires patients to apply rapamycin liquid and placebo liquid twice daily. Participation in the study will take the patient 5 minutes a day, plus the 4 control visits that will take approximately 30 minutes. A potential risk when participating in this study is an allergy for one of the components of rapamune oral solution. Furthermore, local irritation due to the excipient, which contains alcohol, is possible but this can be easily controlled with emollients. There will be one biopsy for patients who have not yet undergone one. A skin biopsy is taken by means of a 3 mm punch after injection of local anaesthetic which is briefly painful. A properly taken biopsy will leave no or minimal scarring. Previous studies have shown that topical application of rapamycin is safe and does not lead to therapeutic plasma levels. The benefit for the patients is in the potential reduction in facial tumour size and number. For BHD patients in general the benefit will be an easily used topical treatment with which fibrofolliculomas may be treated or prevented.

# 1. INTRODUCTION AND RATIONALE

Birt-Hogg-Dubé syndrome (BHD) is a rare autosomal dominant disorder characterized by the occurrence of benign, mostly facial, hair follicle tumours called fibrofolliculoma [1], multiple lung cysts and (probably as a result) spontaneous pneumothorax [2] and an increased renal cancer risk [3-5]. The fibrofolliculomas (FF) can be quite disfiguring and are usually the reason that patients come to medical attention. FF usually appear after the age of 25 years and are progressive. Typically presenting around the nose, they can spread onto the ears, neck and trunk. [1] Although they do not grow beyond 3-4 mm in size, their numbers increase with age so that patients can eventually have hundreds of tumours. Presently, ablative laser is the preferred treatment. In addition, surgical interventions, like excision and shaving, and elektrocoagulation are performed. Disadvantages of these treatments are the risk of complications (scarring, inflammation, hypo-and hyperpigmentation) and recurrence (for laser therapy it is known that FF recur after 2-3 years or even after months [6].) Moreover, these treatments do not prevent the growth of new FF. A topical treatment that is suitable for chronic use and reduces the number of tumours and/or prevents the growth of new ones would be preferable but is not yet available. New insights from genetic and cell biological studies now suggest a potential topical therapy. BHD syndrome is caused by germline mutations in the BHD gene coding for the protein folliculin [7]. Strongly conserved in evolution, folliculin’s function is mostly unknown. We now know, from research on mouse models and our own human data, that in BHD syndrome there likely is deregulation of mTOR signalling [8]. mTOR, mammalian target of rapamycin, is a multiprotein-complex that is a central player in cellular growth regulation and energy sensing [9]. The mTOR pathway consists of two major branches, each mediated by a specific mTOR complex (mTORC). The first complex, mTORC1, controls several pathways that collectively determine the mass (size) of the cell. The second complex, mTORC2 controls the actin cytoskeleton and thereby determines the shape of the cell [10]. mTORC1 and possibly mTORC2 respond to growth factors (insulin/IGF), energy status of the cell, nutrients (amino acids), and stress [10], as seen in the figure below. Thus, BHD syndrome belongs in a larger family of disorders characterized by mTOR deregulation, such as tuberous sclerosis complex (TSC) [9, 11]. In TSC, as in BHD, patients develop facial hair follicle tumours called angiofibroma. These tumours strongly resemble fibrofolliculoma and are increasingly seen as a variant of the latter. Very recent findings indicate that angiofibromas of TSC respond favourably to the mTOR inhibitor rapamycin – they disappear after only a few months of oral rapamycin [12]. The action of rapamycin is as follows. Studies in rapamycin activity have shown that it requires an intracellular co-factor, FKBP12 (FK506-binding protein 12). Rapamycin and FKBP12 together form a complex, FKBP12-rapamycin. It has been demonstrated that this complex binds to and inhibits mTORC1 [13](see figure 1). The exact way in which this happens is not yet clear. However, there is a hypothesis that FKBP12-Rapamycine changes the conformation of the raptor-mTOR complex and thereby strongly destabilizes it [10, 14].

# Oral administration has potential side effects that are not acceptable in the context of BHD syndrome. However, there are several studies showing that topical application is safe and may be a feasible strategy. Rapamycin oral solution 1 mg/ml has already been used off-label for non-related mucosal and skin disorders and was both safe and effective, with no significant systemic resorption, when applied topically [15, 16]. Thus, we have theorized that rapamycin oral solution might be used for the treatment of BHD-associated fibrofolliculomas.

#
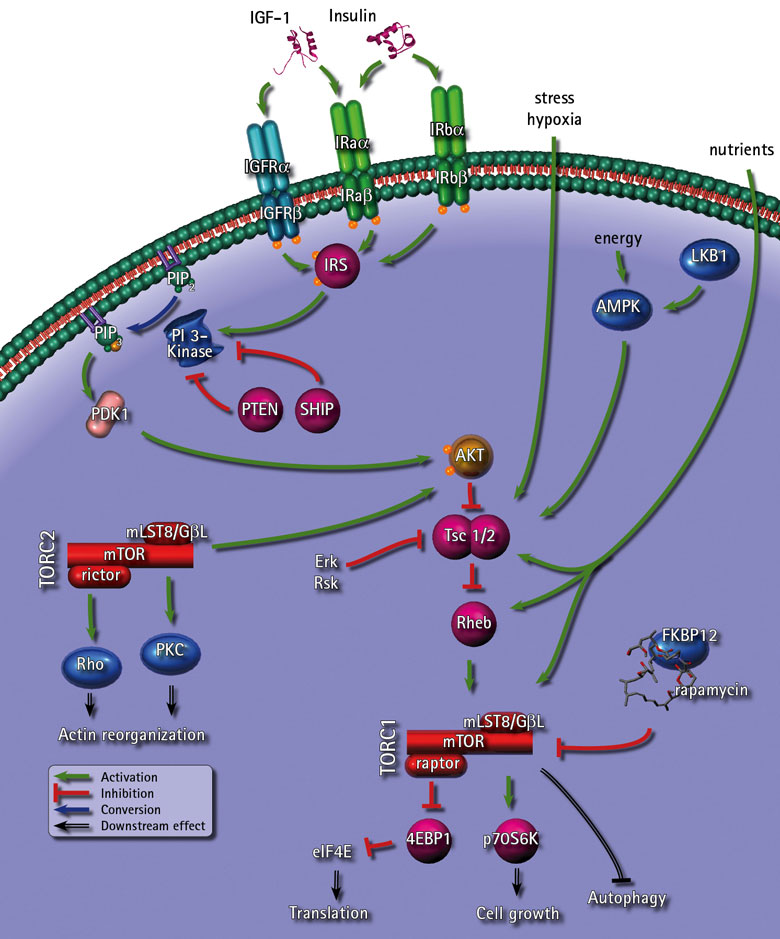


# Figure 1. mTOR pathway

# 2. OBJECTIVES

Primary Objective:

To determine whether topical application of rapamycin 0.1% solution can lead to reduction in size and/or number of fibrofolliculomas in BHD patients and may prevent the growth of new ones.

Secondary Objectives:

To assess safety, formula acceptance and patient satisfaction.

# 3. STUDY DESIGN

The study is double-blind placebo-controlled randomized with the patient him/herself as an internal control. To get enough power we aim to include 20 (see power analysis) adults ≥18 years with Birt-Hogg-Dubé syndrome. Inclusion will take place in two hospitals in the Netherlands; Maastricht University Medical Centre and VU Medical Centre Amsterdam. After inclusion patients are treated with rapamycin in one facial half and a placebo in the other. Patient and physician are blinded until the end of the study. The solution (placebo and verum) is applied according to standardized instructions. The study will proceed according to standardized trial monitor forms (Annex F4). These forms describe how the patient is to be examined at each visit. The skin will be evaluated by 3 observers; the investigator and two dermatologists. They will assess a difference in fibrofolliculoma status on a 7-point rating scale ranging from -3 (strong worsening) to +3 (strong improvement). Although patients evaluate their skin on this scale too, we will not include their measurements in the analysis because of bias. For further explanation see chapter 7.

Patients will be followed-up at 3 months and at 6 months.

It is likely that, during the study, one facial half will improve because of treatment with rapamycin. However, we do not think it is ethical to ask our patients to deal with one improved facial half and one unimproved facial half. Moreover, when patients see a difference between facial sides, they might apply the solution, that they think is the effective one, on both facial sides. To prevent this situation we developed the following study design:

When a clear difference arises between facial sides, blinding will be broken and we will proceed with an open-label study. There are a few conditions to break the blinding, pointed out below.

- Two of three observers have to see a significant difference. This means that there must be an improvement (or worsening, which we don’t expect) of at least 2 points on the 7 point rating scale.
- When there is a significant difference, the blinding will be broken. Then it will also become clear whether the improved side is the rapamycin-treated side. In this case patients are allowed to use rapamycin on the entire face.
- When patients see a difference between the left and right facial side themselves, they can make an earlier appointment.
- To prevent a large proportion of patients form dropping out at an early stage of the study, we aim to continue treatment for at least 3 months. When patients contact us before the end of this period, we will try to motivate them to continue treatment.
- It is also possible that patients see a moderate or strong improvement, while the observers just see a minimal one. Also in this case we will try to motivate patients to proceed with the study for one more month.

In the last two situations described above it will be difficult to convince patients to continue treatment before blinding is to be broken. We think that a good explanation of the importance of the trial and the benefits for the patient, will help to retain patient adherence. Of course we prefer to continue the study as long as possible. However, when a patient really wants to stop treatment due to a clear difference between facial halves, we will break the blinding.

Although we don’t expect it, rapamycin might worsen skin lesions in individual patients. In this situation, the trial will end, just for this specific patient. Another condition to terminate the study prematurely for a specific patient is when it turns out that rapamycin has no effect at all. In our opinion, rapamycin has no effect when no improvement has occurred after 3 months. We base this 3 month period on several clinical studies in which rapamycin was used for treatment of dermatological diseases. In these studies, the effect of rapamycin was visible within 3 months [12, 15-17].

See Annex F4 for a flow chart that gives an overview of the procedures that subjects undergo and a flow chart for patients. Procedures are also described in chapter 7.

# 4. STUDY POPULATION

## 4.1 Population (base)

Otherwise healthy patients (men and women) ≥18 years of age with genetically proven Birt-Hogg-Dubé syndrome and facial fibrofolliculomas.

Patients with Birt-Hogg-Dubé syndrome ascertained in the Netherlands by the European Birt Hogg Dubé consortium. We are aware of 40 families with a total of 100 patients and we are in the process of ascertaining more. Recruitment of the required number of patients (20, see below) should present no problems.

## 4.2 Inclusion criteria

Minimum age of 18 years, facial fibrofolliculomas with at least 10 lesions, entered in a screening program and free of malignancy as determined during screening (already had a baseline MRI or CT-scan in the last 12 months). Otherwise healthy. Able to understand instructions. Fibrofolliculoma diagnosis must be histologically confirmed. Mutation status must be known. For females: not pregnant and willing to use both oral and barrier contraceptives during the treatment period.

## 4.3 Exclusion criteria

Proven or suspected malignancy of skin or other organs. No histological confirmation. Skin lesions other than fibrofolliculomas that might worsen under rapamycin such as active infections. Pregnancy or failure to comply with contraceptive measures. Not able to comprehend instructions. Age under 18 years. No proven mutation. Less than 10 fibrofolliculomas. Planned facial surgery in the treatment period. Concomitant disease requiring systemic immunosuppressive treatment during the trial period or within 30 days before starting therapy. The use of facial topical immunosuppressive treatment or facial topical drugs that interfere with rapamycin during the trial period or within 30 days before starting therapy. Not capable of informed consent. Drug- or alcohol abuse. Tendency to form keloids.

## 4.4 Sample size calculation

Sample size is calculated to detect a difference of 35% in response as measured with a 7-point scale with a power of 90% and a type 1 error, 2 sided, of 0.05. Using McNemar’s chi-square test for matched pairs (each patient serves as his/her own control) we would need 17 patients. Taking into account 15% drop-outs we need to include 20 patients.

# 5. TREATMENT OF SUBJECTS

## 5.1 Investigational product/treatment

Test treatment:

- Rapamune 1 mg/ml
- Active ingredient: rapamycin
- Strength: 0,1%
- Dosage form: oral solution, in this study for topical use
- Manufacturer: Wyeth Pharmaceuticals

After randomisation, patients apply rapamycin 1 mg/ml oral solution (Wyeth) to one predefined skin area on one facial half, twice daily, during 6 months. The other facial half will be similarly treated with a placebo consisting of the excipients only. One of the excipients is propylene glycol which defines the colour (yellowish) and smell of the solution. Because rapamune and placebo solution both contain this substance, the solutions can not be distinguished. For preparing the placebo solution the excipients (polysorbate 80 and Phosal PG 50) will be merged into a container. Later water is added and everything is mixed for 10 minutes. Regarding storage and transport we may state that rapamycine and placebo both will be stored at a temperature of 2-8°C. Bottles will be packed in firm export material (cardboard) so that bottles can impossibly break.

## 5.2 Use of co-intervention

The use of topical immunosuppressants in the face, including tacrolimus and corticosteroids and the use of any other facial dermatological medication that is interacting rapamycin, is not allowed. Topical medication that is used outside the face can be allowed after consultation with the principal investigator. Oral immunosuppressive medication is not allowed. Any other oral medication is only allowed after consulting the principal investigator.

Females should ensure adequate contraception using oral contraceptives and barrier methods. Local surgical procedures including laser surgery are not allowed. If there is a medical necessity for such procedures the patient must discontinue the trial.

# 6. INVESTIGATIONAL MEDICINAL PRODUCT

## 6.1 Name and description of investigational medicinal product

Rapamune 1 mg/ml oral solution (Wyeth).

## 6.2 Summary of findings from non-clinical studies

In our study we apply this solution topically to treat fibrofolliculomas in BHD.

Topical rapamycin has been used before in several studies. These studies have shown that topical rapamycin was effective in mouse models for different dermatological diseases. A study in nude mice bearing subcutaneous, TSC-related tumours, showed that application of topical rapamycin inhibits TCS tumour growth and improves survival compared to the vehicle cohort. The study also demonstrates that 0,4-0,8% rapamycin applied topically penetrates the skin. [17] Topical rapamycin 0,4%-3,6% was effective in a mouse model for irritant dermatitis and 1% topical rapamycin was effective in mouse model of allergic dermatitis.[18]

## 6.3 Summary of findings from clinical studies

Prior clinical studies showed that topical rapamycin is safe and may be effective in some dermatological diseases.

In a randomized double-blind clinical trial, 8% topical rapamycin was effective for the treatment of psoriasis and there was evidence of skin penetration without measurable rapamycin in blood. [15] An open prospective study of 7 women with chronic erosive oral lichen planus (CEOLP) showed that application of topical rapamycin (1 mg/ml) may be effective in some cases, with negligible absorption into blood and minimal side effects. In this study 4 women had complete remission and 2 had partial remission of lesions. Only 1 woman had rapamycin blood levels that were detectable.[16]

A case report describes how treatment with oral rapamycin in a patient with TSC who underwent renal transplantation, reduced facial angiofibromas dramatically.[12]

Summarized, both clinical and non-clinical studies show that topical rapamycin was both safe and effective. Information about Rapamune 1 mg/ml oral solution may be found in the SPC document: Annex D1, D2 and D3.

## 6.4 Summary of known and potential risks and benefits

A potential risk when participating in this study is an allergy for one of the components of rapamune oral solution. We do not foresee any other risks. Prior research has shown that local application is safe and does not lead to significant plasma levels.

A benefit of topical rapamycin is that side effects are negligible. This contrasts with treatments that are used presently, such as laser and surgical interventions. Complications like scar formation, inflammation, hypo- and hyperpigmentation may occur.

Topical application of rapamycin is potentially associated with temporary irritation and a tingling or slightly painful sensation. However, this is likely due to the alcohol in the excipient. These minor side effects can be easily controlled with a fatty ointment.

Other potential benefits are prevention of new tumour growth and reduction in size and/or number of tumours.

## 6.5 Description and justification of route of administration and dosage

The skin tumours are benign. Although cosmetically disturbing and sometimes even disfiguring, we do feel that oral administration of rapamycin cannot be justified because

this mode of administration has potentially severe side effects. A further description of rapamycin solution is given in chapter 5.

## 6.6 Dosages, dosage modifications and method of administration

Patients apply rapamune 1 mg/ml oral solution (Wyeth) to one predefined skin area on one facial half, twice daily. Patients will be instructed in the procedure to avoid dosage artefacts.

The other facial half will be similarly treated with a placebo consisting of the excipient only. Study medication will be provided in bottles fitted with a syringe adapter. A dosing syringe should be used to withdraw the right amount of solution from the bottle. The patient will be instructed how to use the syringe and to wash hands after each application.

For patient instructions see Annex E4.

Sides to be treated will be randomized once, medication will be labelled with study number, patient initials, visit number, date of distribution and facial side to be treated where LEFT and RIGHT are taken to be determined from the patient’s POV.

## 6.7 Preparation and labelling of Investigational Medicinal Product

Prior to start of the study, rapamycin oral solution will be provided by Wyeth pharmaceuticals. ACE Pharmaceuticals in Zeewolde will be responsible for packaging and labelling the study medication. They will also produce the placebo solution. Rapamune and placebo solution will be packaged in amber glass 20 ml bottles. Decanting of rapamune will take place at a temperature of 2-8 °C at nitrogen conditions to preserve solution quality.

As described above the study medication will be delivered in 20 ml bottles, 6 for each facial half, fitted with a syringe adapter. With a dosing syringe patients withdraw 0,25 ml solution twice daily. . Coding of bottles will be according to the randomisation list.

The following information shall appear on the labels:

ALLEEN VOOR GEBRUIK BIJ KLINISCHE STUDIE

**RECHTS** of **LINKS**

Vloeistof, voor uitwendig gebruik.

2x daags aanbrengen op het gezicht.

Zie patiënten informatie voor meer gegevens.

Studienummer: xxxxx

Houdbaarheid na openen: 1 maand

Bewaren in de koelkast (2-8˚C)

Buiten het bereik en zicht van kinderen bewaren.

Because of a 30 day shelf life of an opened bottle, decanting of rapamune - that requires opening bottles – will be done in 6 stages, ones every month. After preparation at ACE Pharmaceuticals the study medication will be sent directly to the patient by post, so treatment can start immediately. In this way we can guarantee a good quality of rapamune, as patients will be out of solution in exactly one month.

## 6.8 Drug accountability

Subjects should return all used and unused medication / bottles to the investigator. Medication will be collected and returned to the pharmacy.

# *7.* METHODS

## 7.1 Study parameters/endpoints

### 7.1.1 Main study parameter/endpoint

The main study parameter is the change in lesion size and count (severity) as measured by the investigator, the patient and two independent dermatologists. For lesion size we use the average size of fibrofolliculomas in millimetres.

Severity is indicated on a 7-point scale:

-3= strong worsening

-2= moderate worsening

-1= minimal worsening

0= no improvement

1= minimal improvement

2= moderate improvement

3= strong improvement

Significant regression is defined as a difference in assessment by at least 2 of 3 observers, with at least +2 points. The observers are the investigator and two independent physicians. Assessment of size and count is made on the basis of medical photos of the face. These photos will be standardized; same distance, background, colour and light profile etc.

Confirmation by visual examination.

Professor M.H. Prins, professor in clinical epidemiology of Maastricht UMC has advised us to use this 7-point scale. Given the fact that there are no previous randomized controlled trials in the BHD syndrome, there is no previously validated scale that we can use for this disease. That is why we have chosen for a 7-point Likert scale. It is known that the Likert scale has good measurement properties and it is used often in trials in which skin changes have to be assessed [19, 20].

During the study we will determine the intra- and inter-observer variability by means of a weighted kappa test. Per patient 2 photographs will be assessed with the 7-point scale; at 3 months and at 6 months. These photographs will be compared with a baseline photo. To measure the inter- and intra observer variability we need at least 3 observers. These observers will be the researcher and two independent physicians. They will assess every photo twice. To prevent bias, the assessments must be at least 2 months apart. Besides evaluation based on photographs, the researcher as well as the patient can assess facial progress during the appointments. These assessments are not included in the analysis, only the results based on the photographs provided by the researcher, physician 1 and physician 2 will be used. This means there will be 6 scores for every patient per follow-up visit. Of these results, the most frequently given score will be used for the analysis.

### 7.1.2. Secondary study parameters/endpoints

Secondary parameters/endpoints:

- The absence of any effect.
- Safety, assessed by tolerance and adverse event profiles.
- Acceptance of study medication by subjects, indicated on a 5-point rating scale:

0= not acceptable

1= somewhat acceptable

2= moderately acceptable

3= acceptable

4= very acceptable

Patient satisfaction is assessed on the basis of 3 statements. Here patients can express to what extent they agree with the statement. For the extent of agreement a 5-point rating scale is used ranging from total disagreement (-2) to total agreement (+2).

See also trial monitor forms: Annex F4

### 7.1.3. Other study parameters

Other study parameters (baseline characteristics) will be presented in a table.

These other parameters are age, sex, count and average size of FF, previous treatment for FF, age of presentation of FF, other medication used during the trial, medical history.

## 7.2 Randomisation, blinding and treatment allocation

Sides to be treated will be randomized according to a computer randomisation program in blocks of 4. An independent statistician will prepare the randomisation list. Rapamycin and placebo will be packaged in identical bottles with a code number according to the randomisation list. Because of participation of two centres, stratification will take place by allocating entire blocks to a centre. The list of randomisation will be kept at a secure place away from the investigator.

Medication is prepared by Wyeth pharmaceuticals and delivered to the pharmacy of VU Medical Centre Amsterdam. There medication will be labelled with study number and facial side to be treated where LEFT and RIGHT are taken to be determined from the patient’s POV. Patient and physician are blinded until the end of the study.

If at any visit a clear difference arises between treated sides the binding will be broken. If better side is the rapamycin treated one, we will continue with an open-label study. If the better side is the placebo-treated side or the study shows that topical rapamycin has no effect at all, the trial will be terminated prematurely.

Besides unblinding in the situation described above, the code may be broken for a particular subject in case of a suspected serious adverse event. In all other situations unblinding will take place after allocating participants to the relevant analyses populations, just before the primary statistical analysis.

## 7.3 Study procedures

Conduct of the study

The first visit (screening) includes:
- In- or exclusion, in case of inclusion:

- Physical examination according to protocol, especially lesion size and count
- Biopsy of a representative tumour (if not yet undergone one)

- The patient already has the patient information; this has been sent before the first visit.

- For women: pregnancy test

The second visit (baseline) includes:

- Physical examination according to protocol

- Standardized medical photography of the face. (All photos will be assessed by the investigator and two independent physicians).
- Instruction of patient (see Annex E4), provide study medication according to randomization

Follow-up visits will take place at 3 months and at 6 months. At each follow-up visit, unused study medication will be taken back and new medication is dispensed. Unused medication is weighed to ensure protocol compliance.

At the first follow-up visit at 3 months it will be assessed if unblinding is needed.

Physical examination and medical photography are part of regular care/medical treatment.

Treatment with rapamycin solution (and placebo solution) and pregnancy tests are part of the study.

See also trial flow-chart: Annex F4.

## 7.4 Withdrawal of individual subjects

Subjects can withdraw from the study at any time for any reason if they wish to do so without any consequences. The investigator can decide to withdraw a subject from the study for urgent medical reasons.

## 7.5 Replacement of individual subjects after withdrawal

To have enough power we need at least 17 patients. When the number of patients drops below 17, we will replace them by new subjects.

## 7.6 Follow-up of subjects withdrawn from treatment

When subjects are withdrawn from treatment they come for a follow-up visit after 3 months. Then follow-up will conduct according to regular care.

## 7.7 Premature termination of the study

It is possible that the study shows that topical rapamycin has no effect or worsens the skin lesions. In that case, we will terminate the study prematurely. Another reason for terminating the study prematurely is when emerging adverse events are of such serious nature that continuation of the trial becomes unacceptable.

If the study is terminated, patients will be followed-up and treated according to accepted standards of care.

# 8. SAFETY REPORTING

## 8.1 Section 10 WMO event

In accordance to section 10, subsection 1, of the WMO, the investigator will inform the subjects and the reviewing accredited METC if anything occurs, on the basis of which it appears that the disadvantages of participation may be significantly greater than was foreseen in the research proposal. The study will be suspended pending further review by the accredited METC, except insofar as suspension would jeopardise the subjects’ health. The investigator will take care that all subjects are kept informed.

## 8.2 Adverse and serious adverse events

Adverse events are defined as any undesirable experience occurring to a subject during a clinical trial, whether or not considered related to the investigational drug. All adverse events reported spontaneously by the subject or observed by the investiga­tor or his staff will be recorded.

When there is a possible adverse event, the trial physician will consult the PI who will then take appropriate action based on his assessment. An example of an adverse event related to the investigational drug (an adverse reaction) is local irritation after application of rapamycin. If this is not allergic, the trial will continue and the patient will get a corticosteroid temporarily. When local irritation is based on an allergic reaction the patient will be excluded.

When a local side effect appears, the affected side of the face will be recorded on the study forms so that it is possible to check afterwards whether the adverse event was caused by rapamycin or the placebo.

A serious adverse event is any untoward medical occurrence or effect that at any dose results in death;

- is life threatening (at the time of the event);
- requires hospitalisation or prolongation of existing inpatients’ hospitalisation;
- results in persistent or significant disability or incapacity;
- is a congenital anomaly or birth defect;
- is a new event of the trial likely to affect the safety of the subjects, such as an unexpected outcome of an adverse reaction, lack of efficacy of an IMP used for the treatment of a life threatening disease, major safety finding from a newly completed animal study, etc.

All SAEs will be reported to the accredited METC that approved the protocol, according to the requirements of that METC.

### 8.2.1 Suspected unexpected serious adverse reactions (SUSAR)

Adverse reactions are all untoward and unintended responses to an investigational product related to any dose administered.

Unexpected adverse reactions are adverse reactions, of which the nature, or severity, is not consistent with the applicable product information (e.g. Investigator’s Brochure for an unapproved IMP or Summary of Product Characteristics (SPC) for an authorised medicinal product).

The sponsor will report expedited the following SUSARs to the METC:

- SUSARs that have arisen in the clinical trial that was assessed by the METC;
- SUSARs that have arisen in other clinical trial of the same sponsor and with the same medicinal product, and that could have consequences for the safety of the subjects involved in the clinical trial that was assessed by the METC.

The remaining SUSARs are recorded in an overview list (line-listing) that will be submitted once every half year to the METC. This line-listing provides an overview of all SUSARs from the study medicine, accompanied by a brief report highlighting the main points of concern.

The sponsor will report expedited all SUSARs to the competent authority, the Medicine Evaluation Board and the competent authorities in other Member States.

The expedited reporting will occur not later than 15 days after the sponsor has first knowledge of the adverse reactions. For fatal or life threatening cases the term will be maximal 7 days for a preliminary report with another 8 days for completion of the report.

Based on clinical studies described before, we don’t expect SUSAR’s.

### 8.2.2 Annual safety report

In addition to the expedited reporting of SUSARs, the sponsor will submit, once a year throughout the clinical trial, a safety report to the accredited METC, competent authority, Medicine Evaluation Board and competent authorities of the concerned Member States.

This safety report consists of:

- a list of all suspected (unexpected or expected) serious adverse reactions, along with an aggregated summary table of all reported serious adverse reactions, ordered by organ system, per study;
- a report concerning the safety of the subjects, consisting of a complete safety analysis and an evaluation of the balance between the efficacy and the harmfulness of the medicine under investigation.

## 8.3 Follow-up of adverse events

All adverse events will be followed until they have abated, or until a stable situation has been reached. Depending on the event, follow up may require additional tests or medical procedures as indicated, and/or referral to the general physician or a medical specialist.

# 9. STATISTICAL ANALYSIS

Professor M.H. Prins from the KEMTA will be responsible for the statistical evaluation of the study results.

## 9.1 Descriptive statistics

Data will be described as mean (+/- standard deviation), median [interquartile range] or percentages as appropriate. Changes in fibrofolliculoma status will be tested using a non-parametric test for paired observations, known as McNemar’s chi square test.

## 9.2 Primary analysis

During the trial patients apply rapamycin solution in one facial half and placebo solution in the other facial half. This means that the patient is his/her own control.

Primary output measure is change in facial fibrofolliculoma size and count (severity) as measured by the investigator, two independent physicians and the patient. The response to treatment will be assessed with a 7-point rating scale, ranging from -3 (strong worsening) to +3 (strong improvement). This scale is described in chapter 7. Significant regression is defined as assessment by at least 2 of 3 observers with at least +2 points. Rapamycin will be compared with placebo using a McNemar ‘s chi square test for paired samples. Both an intention to treat and a per protocol analysis will be performed.

## 9.4 Interim analysis

Not planned.

Rapamycin is a well-known immunosuppressant with known side effects. When it is used topically side effects are negligible. For this reason we do not include an interim analysis.

# 10. ETHICAL CONSIDERATIONS

## 10.1 Regulation statement

The study will be conducted according to principles of the latest revision of the Declaration of Helsinki and in accordance with the Medical Research Involving Human Subjects Act (WMO).

## 10.2 Recruitment and consent

As described above patients with Birt-Hogg-Dubé syndrome ascertained in the Netherlands by the European Birt Hogg Dubé consortium. We are aware of 40 families with a total of 100 patients and we are in the process of ascertaining more. These patients receive their follow-up at the Maastricht University Medical Centre and VU Medical Centre in Amsterdam. Their information has been recorded in databases. These databases will be used to make a selection of patients that may qualify for this study. The selected patients will receive a letter from their doctor, who will be the principal investigator in this case. In this letter the trial will be announced. After they have received the letter they will be called and informed about the study by the investigator. If the patient is interested he/she will receive the patient information by post. Patients will get a consideration period of 2 weeks in which they can choose to participate or not. Patients and subjects can contact an independent physician with any question.

The informed consent form will be signed by the investigator and the subject. A copy of the signed Subject Informed Consent form shall be given to each study subject. The signed Subject Informed Consent form must be kept at the investigational site with the study documentation.

For the patient information letter see Annex E1 and for the informed consent form see Annex E2.

## 10.3 Benefits and risks assessment, group relatedness

Participation requires patients to apply rapamycin liquid and placebo liquid twice daily.

Participation in the study will take the patient 5 minutes a day, plus the control visits every 3 months that will take approximately 30 minutes. As described above, a potential risk when participating in this study is an allergy for one of the components of rapamune oral solution.

Furthermore, local irritation due to the excipient, which contains alcohol, is possible but this can be easily controlled. There will be one biopsy for patients who have not yet undergone one. A skin biopsy is taken by means of a 3 mm punch after injection of local anaesthetic which is briefly painful. A properly taken biopsy will leave no or minimal scarring. If a patient has a known tendency to form keloids or hypertrophic scars, a biopsy is undesirable for cosmetic reasons.

Previous studies have shown that topical application of rapamycin is safe and does not lead to therapeutic plasma levels. The benefit for the patients is in the potential reduction in facial tumour size and number. For BHD patients in general the benefit will be an easily used topical treatment with which fibrofolliculomas may be treated or prevented.

## 10.4 Compensation for injury

The sponsor/investigator has a liability insurance (Lloyd’s of London) which is in accordance with article 7, subsection 6 of the WMO.

The sponsor (also) has an insurance which is in accordance with the legal requirements in the Netherlands (Article 7 WMO and the Measure regarding Compulsory Insurance for Clinical Research in Humans of 23rd June 2003). This insurance provides cover for damage to research subjects through injury or death caused by the study.

1. € 450.000,-- (i.e. four hundred and fifty thousand Euro) for death or injury for each subject who participates in the Research;
2. € 3.500.000,-- (i.e. three million five hundred thousand Euro) for death or injury for all subjects who participate in the Research;
3. € 5.000.000,-- (i.e. five million Euro) for the total damage incurred by the organisation for all damage disclosed by scientific research for the Sponsor as ‘verrichter’ in the meaning of said Act in each year of insurance coverage.

The insurance applies to the damage that becomes apparent during the study or within 4 years after the end of the study.

For further information see the insurance text: Annex G1.

**10.5 Incentives**

Patients will receive travel cost compensation for extra hospital visits made for participating in the study.

# 11. ADMINISTRATIVE ASPECTS AND PUBLICATION

## 11.1 Handling and storage of data and documents

Patient material (study data, medical photos etc.) are stored in a protected computer file and will be processed anonymously. A subject identification code list is used to link the data to the subject. The coding is not based on date of birth. Patient material will be coded in the same way. The key to the data and patient material code both will be safeguarded by the principal investigator.

Data and patient material will be stored after the trial is ended for further future research for a maximum of 15 years. During and after the study, researcher and responsible physician have access to patient material and data.

Patients will be informed and asked for their permission for further future analysis in the informed consent form (Annex E2).

## 11.2 Amendments

A ‘substantial amendment’ is defined as an amendment to the terms of the METC application, or to the protocol or any other supporting documentation, that is likely to affect to a significant degree:

- the safety or physical or mental integrity of the subjects of the trial;
- the scientific value of the trial;
- the conduct or management of the trial; or
- the quality or safety of any intervention used in the trial.

All substantial amendments will be notified to the METC and to the competent authority.

Non-substantial amendments will not be notified to the accredited METC and the competent authority, but will be recorded and filed by the sponsor.

## 11.3 Annual progress report

The sponsor/investigator will submit a summary of the progress of the trial to the accredited METC once a year. Information will be provided on the date of inclusion of the first subject, numbers of subjects included and numbers of subjects that have completed the trial, serious adverse events/ serious adverse reactions, other problems, and amendments.

## 11.4 End of study report

The sponsor will notify the accredited METC and the competent authority of the end of the study within a period of 90 days. The end of the study is defined as the last patient’s last visit.

In case the study is ended prematurely, the sponsor will notify the accredited METC and the competent authority within 15 days, including the reasons for the premature termination.

 Within one year after the end of the study, the investigator/sponsor will submit a final study report with the results of the study, including any publications/abstracts of the study, to the accredited METC and the Competent Authority.

## 11.5 Public disclosure and publication policy

No limitations will be applicable with regard to publication of the results of the study.

The research findings will be published in international peer-reviewed open access journals or will be communicated in (inter)national conferences to scientists / physicians. In exceptional cases, if the public interest so requires, we will spread the results via the popular media.

# 12. REFERENCES

1. Birt, A.R., G.R. Hogg, and W.J. Dube, *Hereditary multiple fibrofolliculomas with trichodiscomas and acrochordons.* Arch Dermatol, 1977. **113**(12): p. 1674-7.

2. Toro, J.R., et al., *Lung cysts, spontaneous pneumothorax, and genetic associations in 89 families with Birt-Hogg-Dube syndrome.* Am J Respir Crit Care Med, 2007. **175**(10): p. 1044-53.

3. Pavlovich, C.P., et al., *Renal tumors in the Birt-Hogg-Dube syndrome.* Am J Surg Pathol, 2002. **26**(12): p. 1542-52.

4. Roth, J.S., et al., *Bilateral renal cell carcinoma in the Birt-Hogg-Dube syndrome.* J Am Acad Dermatol, 1993. **29**(6): p. 1055-6.

5. Toro, J.R., et al., *Birt-Hogg-Dube syndrome: a novel marker of kidney neoplasia.* Arch Dermatol, 1999. **135**(10): p. 1195-202.

6. Gambichler, T., et al., *Treatment of Birt-Hogg-Dube syndrome with erbium:YAG laser.* J Am Acad Dermatol, 2000. **43**(5 Pt 1): p. 856-8.

7. Nickerson, M.L., et al., *Mutations in a novel gene lead to kidney tumors, lung wall defects, and benign tumors of the hair follicle in patients with the Birt-Hogg-Dube syndrome.* Cancer Cell, 2002. **2**(2): p. 157-64.

8. Baba, M., et al., *Kidney-targeted Birt-Hogg-Dube gene inactivation in a mouse model: Erk1/2 and Akt-mTOR activation, cell hyperproliferation, and polycystic kidneys.* J Natl Cancer Inst, 2008. **100**(2): p. 140-54.

9. Baba, M., et al., *Folliculin encoded by the BHD gene interacts with a binding protein, FNIP1, and AMPK, and is involved in AMPK and mTOR signaling.* Proc Natl Acad Sci U S A, 2006. **103**(42): p. 15552-7.

10. Wullschleger, S., R. Loewith, and M.N. Hall, *TOR signaling in growth and metabolism.* Cell, 2006. **124**(3): p. 471-84.

11. Inoki, K., M.N. Corradetti, and K.L. Guan, *Dysregulation of the TSC-mTOR pathway in human disease.* Nat Genet, 2005. **37**(1): p. 19-24.

12. Hofbauer, G.F., et al., *The mTOR inhibitor rapamycin significantly improves facial angiofibroma lesions in a patient with tuberous sclerosis.* Br J Dermatol, 2008. **159**(2): p. 473-5.

13. Jozwiak, J., S. Jozwiak, and M. Oldak, *Molecular activity of sirolimus and its possible application in tuberous sclerosis treatment.* Med Res Rev, 2006. **26**(2): p. 160-80.

14. Foster, D.A. and A. Toschi, *Targeting mTOR with rapamycin: one dose does not fit all.* Cell Cycle, 2009. **8**(7): p. 1026-9.

15. Ormerod, A.D., et al., *Treatment of psoriasis with topical sirolimus: preclinical development and a randomized, double-blind trial.* Br J Dermatol, 2005. **152**(4): p. 758-64.

16. Soria, A., et al., *Treatment of refractory oral erosive lichen planus with topical rapamycin: 7 cases.* Dermatology, 2009. **218**(1): p. 22-5.

17. Rauktys, A., et al., *Topical rapamycin inhibits tuberous sclerosis tumor growth in a nude mouse model.* BMC Dermatol, 2008. **8**: p. 1.

18. Baumer, W., et al., *Cilomilast, tacrolimus and rapamycin modulate dendritic cell function in the elicitation phase of allergic contact dermatitis.* Br J Dermatol, 2005. **153**(1): p. 136-44.

19. Leyden, J.J., et al., *Topical retinoids in inflammatory acne: a retrospective, investigator-blinded, vehicle-controlled, photographic assessment.* Clin Ther, 2005. **27**(2): p. 216-24.

20. Ozolins, M., et al., *Randomised controlled multiple treatment comparison to provide a cost-effectiveness rationale for the selection of antimicrobial therapy in acne.* Health Technol Assess, 2005. **9**(1): p. iii-212.

**13.ANNEXES**

D1, D2 and D3: Investigator’s Brochure and SPC document

E1: Patient information

E2: Informed consent form

E4: Instruction study medication

F4: Flow-chart trial, flow-chart for patients and trial monitor forms

G1: Insurance text
